# Pretreatment Lifestyle Behaviors as Survival Predictors for Patients with Nasopharyngeal Carcinoma

**DOI:** 10.1371/journal.pone.0036515

**Published:** 2012-05-08

**Authors:** Guo-Ping Shen, Feng-Hua Xu, Fen He, Hong-Lian Ruan, Cui Cui, Li-Zhen Chen, Yi-Xin Zeng, Wei-Hua Jia

**Affiliations:** 1 State Key Laboratory of Oncology in South China, Cancer Center, Sun Yat-sen University, Guangzhou, China; 2 Department of Radiation Oncology, The First Affiliated Hospital, Sun Yat-sen University, Guangzhou, China; 3 Department of Radiation Oncology, The First Affiliated Hospital, Guangzhou Medical University, Guangzhou, China; Virginia Commonwealth University, United States of America

## Abstract

**Background:**

Lifestyle behaviors have been widely reported to influence the survival of patients with head and neck cancer. However, the relationship between pretreatment lifestyle behaviors and survival among patients with nasopharyngeal carcinoma (NPC) is unclear.

**Methods:**

A prospective cohort study was designed to determine the relationship between lifestyle behaviors and survival in 1,533 NPC patients recruited from October 2005 to October 2007. Pretreatment lifestyle behaviors (such as body-mass index [BMI], smoking, alcohol, diet) of the patients were investigated. Univariate and multivariate proportional-hazards models were used to assess the impact of lifestyle behaviors on patient survival.

**Results:**

Smoking was a predictor of survival; both current smokers (hazard ratio [HR] = 1.88; 95% CI, 1.33 to 2.65) and heavy smokers (≥25 Pack-years; HR = 1.84; 95% CI, 1.30 to 2.60) showed associations with poor survival. Higher BMI was significantly associated with a lower risk of death (*P*_trend_ = 0.002). Compared with under/normal-weight patients (BMI less than 22.99 kg/m^2^), the multivariate HR for survival was 0.66 (95% CI, 0.48 to 0.90) and 0.47 (95% CI, 0.23 to 0.97) for overweight and obese patients, respectively. No alcohol intake and high fruit intake were associated with favorable survival in the univariate analysis but lost significance in the multivariate model.

**Conclusion:**

Our findings indicate that pretreatment lifestyle behaviors, especially smoking status and BMI, as easily available data, provide prognostic value for survival in NPC patients.

## Introduction

Lifestyle behaviors are reported to influence clinical outcomes for patients with head and neck cancer [1]–[3]. In a multicenter study, cigarette smoking and high levels of alcohol consumption were found to adversely affect the survival of patients with laryngeal and hypopharyngeal cancers, whereas high intakes of vegetables and poultry had favorable effects on prognosis [2]. Another prospective cohort study found that pretreatment health behaviors (e.g., smoking, fruit intake, and physical activity) predicted survival among patients with head and neck squamous cell carcinoma [3].

Nasopharyngeal carcinoma (NPC) is a unique and endemic head and neck epithelial malignancy that occurs predominantly in southern China. Epstein Barr virus (EBV) being a primary cause of NPC plays an important role on the etiology [4]. Lifestyle behaviors such as smoking tobacco, consuming alcohol, eating salted-fish and other dietary habits have been linked to the development of NPC [5]–[7]. However, whether lifestyle behaviors are associated with the survival of patients with NPC is unclear.

In previous research, we conducted an epidemiological investigation to evaluate lifestyle and other environmental factors influencing NPC risk. In that study, our team profiled the lifestyle behaviors of newly identified NPC patients at diagnosis. Simultaneously, we launched a prospective cohort study to assess the association between lifestyle behaviors and survival in NPC patients. Now, after longer follow-up, the present study was undertaken to determine whether lifestyle behaviors (including body-mass index [BMI], smoking, alcohol use, dietary factors, tea consumption, and a Cantonese herbal tea habit) predict survival among patients with NPC.

If pretreatment lifestyle behaviors are predictive of survival among patients with NPC, the information would be useful for developing clinical and public health interventions to improve survival. The lifestyle behaviors questionnaire may become a practical and non-invasive means of identifying those patients at high risk who require tailored medical support before treatment and those who must be observed more closely after treatment.

Moreover, lifestyle behaviors not only associated with increased risk for certain cancers and survival period, but also with enhanced risk of chronic diseases such as diabetes, stroke, and coronary heart disease, all of which influence on people's life length severely [8], [9]. From the point of view, investigation on lifestyle behaviors could be great meaningful price for public health.

## Results

The demographic characteristics of the patients, their lifestyle behaviors, and their clinical information are all summarized in Table 1. At the time of diagnosis, the mean patient age was 46.1 years (SD = 11.1 years). Most patients were living with their spouse (94.7%). Four-hundred-ten patients (26.7%) were female and 234 patients (15.3%) had higher education. Approximately one-half smoked, one-third drank regularly, and almost one-quarter consumed fruit less than once per month. The mean BMI was 22.6 kg/m2 (SD = 3.2 kg/m^2^). one hundred and forty-one patients (9.2%) were underweight, 733 patients (47.8%) were normal-weight, 552 patients (36%) were overweight and 107 patients (7%) were obese. After a median follow-up of 3.3 years, 243 deaths (15.9%) were observed among the 1,533 participants. The 3-year survival rate was 86.8% (95% CI, 85.0%–88.5%).

**Table 1:** Patient characteristics.

Variable	No (n = 1533)	%
**Age, years**		
Mean	46.1	
SD	11.1	
≤30	108	7.1
31–40	385	25.1
41–50	512	33.4
51–60	367	23.9
≥61	161	10.5
**Sex**		
Male	1123	73.3
Female	410	26.7
**Marital status**		
With spouse	1451	94.7
Without spouse	82	5.3
**Educational level**		
High school or less	1299	84.7
University or more	234	15.3
**Body mass index, kg/m^2^**		
Mean	22.6	
SD	3.2	
Underweight (less than 18.5)	141	9.2
Normal weight (18.5 to 22.99)	733	47.8
Overweight (23.0 to 27.49)	552	36.0
Obesity (27.5 or more)	107	7.0
**Smoking status**		
Current	436	28.4
Former	395	25.8
Never	702	45.8
**Pack-years**		
Never	712	46.4
<25 Pack-years	437	28.5
≥25 Pack-years	384	25.0
**Alcohol intake**		
Nondrinker	912	59.5
≤1 drink per day	533	34.8
>1 drink per day	88	5.7
**Duration of alcohol intake**		
Nondrinker	912	59.5
<20 years	533	34.8
≥20 years	88	5.7
**Fruits**		
Less than monthly	353	23.0
Monthly∼weekly	645	42.1
Daily or more	535	34.9
**Clinical stage**		
I	106	6.9
II	350	22.8
III	614	40.1
IV	463	30.2
**Pathology**		
WHO I	19	1.2
WHO II	132	8.6
WHO III	1382	90.2
**Treatment**		
Radiotherapy alone	440	28.7
Radiotherapy+chemotherapy	456	29.7
Concurrent chemoradiotherapy	637	41.6

### Univariate Cox Proportional Hazards Regression Analysis

Clinical variables found to be associated (P<0.05) with survival in the univariate Cox proportional hazards regression analyses included age (1-year increment, P = 0.004), sex (female vs. male, P<0.0001), clinical stage (P<0.0001), and treatment (concurrent chemoradiotherapy versus radiotherapy plus induction and/or adjuvant chemotherapy, P<0.0001). Among the two demographic characteristics (marital status and education), being without a spouse was not associated with survival in univariate analysis. A favorable survival was found to be associated with higher education (P = 0.008) (table 2).

**Table 2:** Univariate hazard ratios from Cox proportional hazards regression models.

Variable	HR	95% CI	*P*
Age (≥v<46 years)	1.41	1.09 to 1.81	.009
Female	0.51	0.37 to 0.71	<.0001
Clinical stage	1.86	1.58 to 2.20	<.0001
Treatment (CRT *v* RT+CT)	0.47	0.35 to 0.63	<.0001
Without spouse	1.21	0.69 to 2.12	.507
University or more	0.57	0.38 to 0.86	.008
Smoking status			*P*_trend_<.0001
Never smoked	1.00	Ref.	
Former smoker	2.06	1.49 to 2.85	<.0001
Current smoker	2.14	1.57 to 2.90	<.0001
Pack-years			*P*_trend_<.0001
Never	1.00	Ref.	
<25 Pack-years	1.85	1.34 to 2.54	<.0001
≥25 Pack-years	2.46	1.80 to 3.37	<.0001
Alcohol intake			*P*_trend_ = .001
Nondrinker	1.00	Ref.	
≤1 drink per day on average	1.26	0.95 to 1.65	.104
>1 drink per day on average	2.22	1.41 to 3.50	.001
Duration of alcohol intake			*P*_trend_ = .009
Nondrinker	1.00	Ref.	
<20 years	1.28	0.98 to 1.67	.070
≥20 years	1.81	1.10 to 2.98	.019
Frequency of fruit intake			*P*_trend_ = .008
Less than monthly	1.00	Ref.	
Monthly - weekly	0.77	0.56 to 1.05	.097
Daily or more	0.63	0.46 to 0.89	.007
BMI (kg/m^2^)			*P*_trend_ = .002
Underweight (18.48 or less)	0.92	0.59 to 1.44	.724
Normal (18.50 to 22.99)	1.00	Ref.	
Overweight (23.00 to 27.49)	0.62	0.46 to 0.85	.003
Obese (27.50 or more)	0.44	0.22 to 0.90	.025

Abbreviation: HR, Hazard Ratio; CRT, Concurrent chemoradiotherapy; RT+CT, Radiotherapy+Chemotherapy.

**Figure 1:**
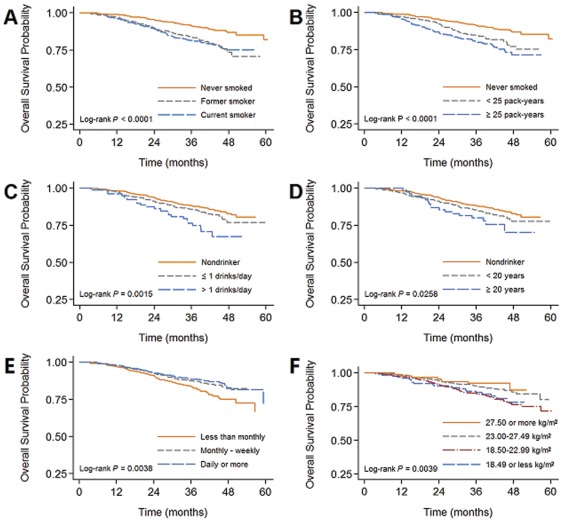
Kaplan-Meier plots of the influence of various lifestyle behaviors on survival in NPC patients. (A) Smoking status; (B) Lifetime cigarette consumption; (C) Alcohol intake; (D) Duration of alcohol intake; (E) Frequency of fruit intake; (F) Body-mass index.

The univariate analysis showed that four lifestyle behaviors (smoking status, alcohol intake, fruit intake, and BMI) were significantly associated with survival (Fig. 1). The pretreatment smoker, whether current or former smoker, were strongly associated with poor survival. A pack-years dosage effect on survival was observed when pack-years were divided into light smokers (<25 pack-years; HR = 1.85; 95% CI, 1.34 to 2.54) and heavy smokers (≥25 pack-years; HR = 2.46; 95% CI, 1.80 to 3.37) in the univariate analysis (P_trend_<0.0001). Frequency of alcohol intake was also associated with poor survival. A strong adverse effect on survival was observed with an average of more than 1 drink per day, with a hazard ratio of 2.22 (95% CI, 1.41% to 3.50%, P = 0.001). A drinking duration of 20 years or more was also associated with unfavorable survival (P = 0.019). High frequency intake of fruit significantly improved the survival. Daily consumption of fruit was associated with 37% (95% CI, 11% to 54%) reduction in the likelihood of death. Being overweight or obese was positively associated with survival. The risk of death was 0.62 (95% CI, 0.46 to 0.85) and 0.44 (95% CI, 0.22 to 0.90) for overweight and obese individuals, respectively, compared with those of normal weight. The results of the univariate Cox proportional hazards regression models are listed in Table 2. However, we did not find that consumption of Cantonese herbal tea or tea was associated with survival (Table S1).

### Multivariate Cox Proportional Hazards Regression Analysis

We developed a multivariate model to adjust the association between lifestyle behaviors and survival (table 3). Considering the multicollinearity among lifestyle behavior variables, the variables in multivariate model included age, marital status, education, clinical stage (3 levels), smoking (current, former, and never), alcohol drink (>1/day vs. <1/day), fruits (3 levels), and BMI (3 levels). The correlation coefficients of the association between each variable were all not greater than 0.3. The multivariate model was not included the variable of sex because the coefficient of correlation between sex and smoking status was 0.59 (>0.30). Treatment was also not included as a covariate in model because the patients were generally treated with guideline base on their cancer stage and treatment was inextricably associated with tumor stage and the coefficient of correlation between treatment and clinical stage was 0.39 (>0.30). Of the lifestyle behaviors, smoking status and BMI remained statistically significant. The pretreatment current smoker variable was associated with poor survival. The risk of death was 1.88 (95% CI, 1.33 to 2.65) and 1.66 (95% CI, 1.14 to 2.41) for current and former smokers versus non-smokers, respectively. BMI was associated with survival in multivariate model. As BMI increased from underweight to obese, there was a trend towards a reduction in the likelihood of death in multivariate analysis (*P*_trend_ = 0.002). Compared with normal-weight or underweight patients, the multivariate HR for survival was 0.66 (95% CI, 0.48 to 0.90) and 0.47 (95% CI, 0.23 to 0.97) for overweight and obese patients, respectively. When variable of pack-years replaced smoking status The pretreatment light smoker was found a 68% (95% CI, 15% to 146%) increase in the likelihood of death and higher likelihood of death was 84% (95% CI, 30% to 160%) for heavy smoker. The dosage effect of pack-years on survival was significant in multivariate analysis (*P*_trend_ = 0.003). Of the other lifestyle behaviors, alcohol intake and frequency of fruit intake lost statistically significant associations with survival after adjustment for clinical variables and demographic characteristics.

**Table 3:** Multivariate analyses of clinical variables, demographics and lifestyle behaviors to predict survival.

Variables	HR	95% CI	*P*
Age (≥v<46 years)	1.26	0.94 to 1.69	0.122
Without spouse	1.59	0.87 to 2.92	0.135
University or more	0.86	0.56 to 1.32	0.486
Clinical stage			*P*_trend_<0.001
Stage I+II	1.00	Ref.	
Stage III	2.47	1.60 to 3.81	<0.001
Stage IV	3.11	2.02 to 4.78	<0.001
Smoking status			*P*_trend_<0.001
Never smoked	1.00	Ref.	
Former smoker	1.66	1.14 to 2.41	0.008
Current smoker	1.88	1.33 to 2.65	<0.001
Alcohol intake			
≤1 drink per day on average	1.00	Ref.	
>1 drink per day on average	1.28	0.77 to 2.14	0.341
Frequency of fruit intake			*P*_trend_ = 0.212
Less than monthly	1.00	Ref.	
Monthly - weekly	0.84	0.60 to 1.20	0.341
Daily or more	0.78	0.53 to 1.14	0.203
BMI (kg/m^2^)			*P*_trend_ = 0.002
Underweight or normal	1.00	Ref.	
Overweight	0.66	0.48 to 0.90	0.010
Obese	0.47	0.23 to 0.97	0.041

Note: Each HR is adjusted simultaneously for all the other variables in the table.

Abbreviation: HR, Hazard Ratio.

## Discussion

A number of studies have reported that smoking is a predictor of unfavorable survival among head and neck cancer. Most of these studies have shown that smoking before treatment was associated with a worse prognosis [1]–[3], [10], [11]. A few of the studies reported that the survival of patients with head and neck cancer who continued to smoke during therapy was lower than that of patients who quit smoking [12]–[14]. Whether head and neck squamous cell carcinoma are caused by smoking or not, pretreatment smoking has been reported to influence those patients' survival. However, NPC is rare in most part of world, few or no cases of NPC were included in the study populations in previous reports. To date, whether or not smoking influences the survival of patients with NPC is unknown. The large prospective study described here is novel because the cohorts were limited to a specific disease, NPC, with a unique etiology and unique patient characteristics. Our findings provide evidence that pretreatment smoking is associated with a worse prognosis in patients with NPC. The pretreatment smoking status record may provide an easily available, practical, and non-invasive supplement for predicting clinical outcomes. This information would also offer clinicians guidance for early intervention and follow-up more closely after treatment in heavy smokers.

Heavy alcohol use might lead to nutritional deficiencies by reducing the consumption of foods rich in micronutrients [15]. In addition, alcohol consumption can reduce immune surveillance [16]. thus favoring cancer development. The suppression of natural killer cell activity in alcohol-exposed mice lends support to this idea [17]. In this study, Pretreatment alcohol intake was associated with poor survival in univariate analysis but was not a predictor of survival in multivariate analysis. Our findings are consistent with the role of alcohol reported in a study by Duffy et al. These authors found that the relationship between drinking problem and the survival of patients with head and neck cancer was significant in univariate analysis but not in multivariate analysis [3]. Similarly, Dikshit et al. observed that only high consumption of alcohol (≥121 g/day) had a marginally significant influence on overall survival in laryngeal and hypopharyngeal cancers (HR = 1.3; 95% CI, 1.0 to 1.6) [2]. Farshadpour et al. reported that only tobacco and alcohol used together can affect overall survival in patients with head and neck squamous cell carcinoma [18]. In several case-control studies, alcohol consumption was not associated with NPC risk in Asian populations [19]–[22]. Here, we present additional data describing the relationship between pretreatment alcohol intake and outcome in nasopharyngeal carcinoma survivors. The effects of continued alcohol use after diagnosis or treatment on survival of NPC patient deserved the collection of data to describe.

The pretreatment intake of fruit was significantly related to longer survival time in NPC patients in univariate analysis. A survival advantage associated with fruit consumption was also identified among patients with head and neck squamous cell carcinoma in univariate analysis [3]. Fruits are abundant sources of anti-oxidants from multi-vitamins that may protect against cancer [23]. Anti-oxidants counter the effects of reactive oxygen species, compounds that initiate lipid peroxidation, leading to cell damage, disruption of cell signaling, and increased viral replication and expression, which are all pivotal events in carcinogenesis [24]–[27]. A vegetable-rich diet may have a favorable effect on cancer prognosis (e.g., breast cancer, colon cancer, epithelial ovarian cancer, and oral cancer) [25], [28]–[31]. In addition, we found that pretreatment consumption of neither tea nor Cantonese herbal tea influenced the survival of patients with NPC.

Increased body weight was associated with increased death rates for most cancers [32]. But an association between low BMI at diagnosis and poor prognosis has sometimes also been reported in patients with lung cancer [32], [33], head and neck cancer [34], breast cancer [35], [36], colorectal cancer [37], and pancreatic cancer [38]. There are several possible reasons underlying an association between low weight and poor outcome in different cancers. The further analysis was found that the NPC patients with normal/low BMI were more likely to have an advanced stage at diagnosis (Table S4). This was interesting that the consistent finding was reported in esophageal cancer [39]. It helped us to explain overweight/obese NPC patients obtained better survival partially due to those with early stage. However, we have already adjusted clinical staging in multivariate model, and the BMI remained an independent prognostic factor. This results need to be further investigated by other independent cohorts. Another explanation we speculated was that a patient with a higher pretreatment BMI might have more weight to lose and fall to normal weight during treatment than would a patient of normal weight. The pretreatment overweight and obese patients might show strong resistance to malnutrition. In some cases, the treatment of an NPC patient is interrupted often due to malnutrition during radiotherapy [40]. As we know, interruptions or prolonged treatment have an adverse effect on radiotherapy outcomes in NPC patients [41]. Moreover, Fang et al found that pretreatment nausea/vomiting and appetite loss were predictive of poor overall survival after adjusting for age, sex, smoking, and cancer stage in multivariate analysis [42]. It supported our finding that decreased BMI was associated with poor survival in patients with NPC. Unfortunately, for the budget limitation, we haven't obtained the information of weight lost during treatment. The impact of change in weight during treatments could not be assessed.

There are some limitations in our study. First, several studies referred that the occurrence of lifestyle changes during lifetime (before and after 50 years old) could influence on survival. However, we haven't collected the specific information and the relevant results cannot be provided by this study. Second, lifestyle sociologic public health variables common in western countries, such as mental depression, have not been measured in the study. Third, co-morbidity and quality of life (QoL) are two potential prognostic predictors for patients with NPC. However, until now no significant results have been reported in NPC patients that co-morbidity influence on NPC patients' survival [42]–[44]. In 2010, Fang *at el* have reported that the pretreatment QoL provided predictive value for prognosis of patients with NPC [42]. Our questionnaire was not included the items of co-morbidity score and QoL score. Without adjusting for these two covariates, an explanation for any single lifestyle behavior effect on NPC prognosis should be made carefully.

Recently, more and more prospective cohort studies have provided important evidence of the effect of pretreatment health behaviors on survival. However, as Gritz et al [45] commented, most clinical trials fail to assess status after registration, or fail to analyze these data even if they are collected, therefore, we are missing major opportunities to detect the effects of the behaviors on cancer survivor. To take smoking habit for example, we are still lack of the knowledge of possible adverse effects of smoking and benefits of cessation during the course of treatment and across follow-up [46]. It might be of particular interest to study in patients treated with chemotherapy, given that smoking status is proving to affect differentially the response to some treatment agents via molecular alterations and effects on drug metabolism [47]. Interestingly, Duffy et al [3] have conducted a clinical trial in Head and Neck Cancer, and participants were resurveyed every 3 months for 2 years and annually thereafter, we expect this study could provide very useful information in clinical practice.

In conclusion, to our knowledge, this is the first large prospective study investigating the roles of lifestyle behaviors as predictors of survival for patients with NPC. Our results show that pretreatment smoking and BMI influence the survival of patients with NPC. Moreover, pretreatment alcohol consumption and fruit intake have potential effect on prognosis of NPC survivors. Future assessments as to whether changes in lifestyle behaviors after diagnosis can improve the survival of NPC patients need to be performed in a long-term follow-up study.

## Materials and Methods

### Patients

This was a prospective cohort study. Research assistants approached 1,610 newly diagnosed patients with NPC who were treated at the Sun Yat-sen University Cancer Center (SYSUCC) between October 2005 and October 2007. The exclusion criteria were as follows: age less than 18 years (n = 8); pregnant (n = 0); not treated with radiotherapy (n = 15); previous or synchronous malignancies (n = 1); no contact information for follow-up (n = 40); self-reported as non-Guangdong Chinese and residing outside of Guangdong province (n = 2); unable to understand (n = 1) or unwilling to complete (n = 10) the questionnaire. There were 1,533 patients recruited into the study. The pretreatment evaluation included a physical examination, a chest x-ray, magnetic resonance image scans of the head and neck, a bone scan and an abdominal sonogram.

A structured questionnaire was administered to study participants during face-to-face interviews. Data were collected for demographic characteristics (such as age, gender, marital status, education), lifestyle information (body mass index, cigarette smoking, alcohol intake, dietary habits, tea consumption, and Cantonese herbal tea consumption). After calculation of their body-mass index, the patients were grouped into four BMI classifications according to the WHO recommendations for Asian populations [48]: less than 18.5 kg/m^2^ = underweight; 18.5–22.99 kg/m^2^ = normal-weight; 23.0–27.49 kg/m^2^ = overweight and 27.5 kg/m^2^ or more = obese. The lifestyle questionnaire has been described previously [49], [50]. The smoking items design was based on the Arizona Smoking Assessment Questionnaire [51]. Smoking information included age at initiation and cessation of smoking and the number of cigarettes smoked per day in certain phases of life. Former smokers were defined as smokers who had quit smoking at least 1 year before presentation. Never smokers were defined as those who had smoked fewer than 100 cigarettes in their lifetime. To assess cumulative exposure, pack-years were calculated as follows: the average number of cigarettes smoked per day was divided by 20 and multiplied by the total number of years of reported smoking. Alcohol consumption was measured by reference to the Lifetime Drinking History [52] and Arizona Food Frequency Questionnaire [51]. The consumption of alcohol was recorded as the average amount of all alcoholic beverages (beer, wine, and liquor) that participants consumed during each time period in which their regular drinking were consistent. A medium serving size (one drink) was defined as 12 ounces of beer, 5 ounces of wine, or 1.5 ounces of liquor [53]. The dietary intake frequency (including fruits and vegetables) was assessed by reference to the Arizona Food Frequency Questionnaire. However, vegetables play an important role at the Cantonese dining table. Most Cantonese eat vegetables every day, at lunch, supper, and morning tea (breakfast). In our vegetable data, ninety-nine percent of patients selected the options of eating vegetables every day and at least one dish per meal. Thus, an assessment of the association between vegetable intake and the survival of patients with NPC could not be performed. The Arizona Tea Questionnaire [51] was referenced to estimate tea drinking and Cantonese herbal tea consumption.

Clinical stages were classified using the American Joint Committee on Cancer (AJCC) staging system published in 2002 [54]. The distribution of subjects by clinical stages was as follows: stage I, 106 (6.9%); stage II, 350 (22.8%); stage III, 614 (40.1%); and stage IV, 463 (30.2%). All patients were treated with definitive radiation therapy. Of these, 1,305 patients (85.1%) received two-dimensional conformal RT and 228 patients (14.9%) received intensity-modulated RT. The technical details of the radiation therapy have been described previously [55], [56]. The prescribed dose ranged from 68 to 78 Gy depending on the tumor stage, with a daily fraction of 2.0 to 2.27 Gy and five fractions per week. More than two-thirds of the patients received combination chemoradiotherapy because of their advanced stage; 637 patients (41.6%) received concurrent chemotherapy, and 456 patients (29.7%) received induction and/or adjuvant chemotherapy. The prescribed concurrent chemotherapy regimen was 80 to 100 mg/m^2^ of cisplatin every 3 weeks or 30 to 40 mg/m^2^ of cisplatin every week. The induction or adjuvant chemotherapy regimen was fluorouracil plus cisplatin-based and was administered every 3 weeks.

### Follow-up

After completion of therapy, the patients were observed regularly until death or their last follow-up appointment. For each subject, the start of the follow-up period was defined as the date of diagnosis of nasopharyngeal carcinoma. The end of the follow-up period was set to 31 October 2010. The patients were observed at 3-month intervals during the first 3 years and at 6-month intervals thereafter. For those patients who were lost to follow-up, their survival information was obtained from Death Registry in the Department of Public Security of guangdong province. Patients lost to follow-up and not found on the Death Registry File were censored at the last follow-up appointment. The median follow-up duration was 40 months (range, 2.07 to 60.43 months).

### Statistical analysis

Statistical analysis was performed using the Stata 10.0 statistical software package (Stata Corp.; College Station, TX). Survival time was calculated from the date of NPC diagnosis to the date of death or last follow-up. Those patients who were alive at the last day of follow-up were censored. Median follow-up time was computed with censored observations only. Survival curves were estimated by the Kaplan-Meier method. The log-rank test was used to estimate the statistical differences between survival curves. Cox proportional hazards analysis was performed to calculate the hazard ratio (HR) and the 95% confidence interval (CI) to evaluate the association between lifestyle behaviors and survival. In addition, a multivariate Cox regression was performed to adjust for other covariates. Each lifestyle behavior was considered separately in the Cox proportional hazards regression model because of the multicollinearity among these variables (Table S2 and S3). To assess collinearity in each lifestyle behavior variable, Spearman's correlation was calculated and the variance inflation factor was estimated by multivariate regression [57]–[59]. A two-tailed *P* value of 0.05 or less was considered statistically significant.

### Ethics Statement

This study was reviewed and approved by the Human Ethics Approval Committee of SYSUCC (No. YP2008075). All patients signed informed consent before demographic and lifestyle behavior data were collected by trained SYSUCC staff interviewers.

## Supporting Information

Table S1 Univariate analysis of the influence of tea intake on overall survival.
(DOC)

Table S2 *P*-values of the Spearman correlations among lifestyle behaviors.
(DOC)

Table S3 The variance inflation factors of different lifestyle behaviors.
(DOC)

Table S4 The trend of the early stage NPC patients with higher Body-mass index.
(DOC)
