# Supplementary material for: Pretreatment Lifestyle Behaviors as Survival Predictors for Patients with Nasopharyngeal Carcinoma
Source: PLoS One. 2012 May 8;7(5):e36515. doi: 10.1371/journal.pone.0036515 (PMC3348163; doi:10.1371/journal.pone.0036515)
Supplement: Table S1 — Univariate analysis of the influence of tea intake on overall survival. (DOC) [file pone.0036515.s001.doc]

| Table S1. Univariate analysis of the influence of tea intake on overall survival. | | | | | |
| --- | --- | --- | --- | --- | --- |
| Variable | No (n=1533) | % | Univariate Model | | |
|  |  |  | HR | 95% CI | *P* |
| Cantonese herbal tea consumption |  |  |  |  | *P*_trend_ = .288 |
| Less than monthly | 922 | 60.1 | 1.00 | Ref |  |
| Monthly - weekly | 572 | 37.3 | 0.75 | 0.55 to 1.05 | .092 |
| Daily or more | 39 | 2.5 | 1.24 | 0.58 to 2.64 | .583 |
| Frequency of tea consumption |  |  |  |  | *P*_trend_ = .908 |
| Less than monthly | 823 | 53.7 | 1.00 | Ref. |  |
| Monthly - weekly | 75 | 4.9 | 0.72 | 0.35 to 1.48 | .374 |
| Daily or more | 635 | 41.4 | 1.02 | 0.78 to 1.33 | .884 |
| Type of tea |  |  |  |  |  |
| None | 550 | 35.9 | 1.00 | Ref |  |
| Green tea (daily) | 232 | 15.1 | 1.10 | 0.73 to 1.66 | .647 |
| Oolong tea (daily) | 363 | 23.7 | 0.91 | 0.63 to 1.32 | .633 |
| Black tea (daily) | 100 | 6.5 | 1.49 | 0.90 to2.44 | .118 |
| Pu’er tea (daily) | 147 | 9.6 | 1.03 | 0.63 to 1.70 | .894 |
| Abbreviation: HR, Hazard Ratio. | | | | | |
