# Supplementary material for: Pretreatment Lifestyle Behaviors as Survival Predictors for Patients with Nasopharyngeal Carcinoma
Source: PLoS One. 2012 May 8;7(5):e36515. doi: 10.1371/journal.pone.0036515 (PMC3348163; doi:10.1371/journal.pone.0036515)
Supplement: Table S2 — P-values of the Spearman correlations among lifestyle behaviors. (DOC) [file pone.0036515.s002.doc]

| Table S2. *P*-values of the Spearman correlations among lifestyle behaviors. | | | | | |
| --- | --- | --- | --- | --- | --- |
|  | Smoking status | Pack-years | Alcohol intake | Alcohol duration | Fresh fruits |
| Pack-years | <0.0001 |  |  |  |  |
| Alcohol intake | <0.0001 | <0.0001 |  |  |  |
| Alcohol duration | <0.0001 | <0.0001 | <0.0001 |  |  |
| Fresh fruits | <0.0001 | <0.0001 | 0.0753 | 0.1399 |  |
| BMI | 0.0846 | 0.0981 | 0.0064 | 0.0060 | 0.0020 |
