# Supplementary material for: Pretreatment Lifestyle Behaviors as Survival Predictors for Patients with Nasopharyngeal Carcinoma
Source: PLoS One. 2012 May 8;7(5):e36515. doi: 10.1371/journal.pone.0036515 (PMC3348163; doi:10.1371/journal.pone.0036515)
Supplement: Table S3 — The variance inflation factors of different lifestyle behaviors. (DOC) [file pone.0036515.s003.doc]

| Table S3. The variance inflation factors of different lifestyle behaviors. | | |
| --- | --- | --- |
| Variable | VIF | 1/VIF |
| Smoking status | 6.07 | 0.165 |
| Pack-years | 6.06 | 0.165 |
| Alcohol intake | 8.11 | 0.123 |
| Alcohol duration | 8.11 | 0.123 |
| Fresh fruits | 9.84 | 0.102 |
| BMI | 11.32 | 0.088 |
| Mean VIF | 9.90 |  |
