# Supplementary material for: Pretreatment Lifestyle Behaviors as Survival Predictors for Patients with Nasopharyngeal Carcinoma
Source: PLoS One. 2012 May 8;7(5):e36515. doi: 10.1371/journal.pone.0036515 (PMC3348163; doi:10.1371/journal.pone.0036515)
Supplement: Table S4 — The trend of the early stage NPC patients with higher Body-mass index. (DOC) [file pone.0036515.s004.doc]

| Table S4. The trend of the early stage NPC patients with higher Body-mass index. | | | | |
| --- | --- | --- | --- | --- |
| Variables | Underweight or normal  (BMI < 23 kg/m^2^) | Overweight or obese  (BMI ≥ 23 kg/m^2^) | Odd ratio (95 CI) | *P*-value |
| Clinical stage |  |  |  | *P*_trend_ = 0.002 |
| I | 46 (43.4) | 60 (56.6) | Ref. |  |
| II | 190 (54.3) | 160 (45.7) | 0.65 (0.41 to 1.02) | 0.059 |
| III | 355 (57.8) | 259 (42.2) | 0.56 (0.37 to 0.86) | 0.008 |
| IV | 282 (60.9) | 181 (39.1) | 0.49 (0.32 to 0.77) | 0.002 |
| Note: *P*-value was calculated by logistic regression. | | | | |
